# Coix Seed Oil Ameliorates Rheumatoid Arthritis by Modulating Inflammation-Associated Metabolic Pathways

**DOI:** 10.3390/cimb48050487

**Published:** 2026-05-08

**Authors:** Yong Yang, Ying Feng, Weijie Tang, Yu Meng, Xiuping Ma

**Affiliations:** 1 School of Pharmacy, Guizhou University of Traditional Chinese Medicine, Guiyang 550025, China; yangyong1@gzy.edu.cn (Y.Y.); fengying@stu.gzy.edu.cn (Y.F.); weijietangmr@163.com (W.T.); wm6168913@163.com (Y.M.); 2 The Key Laboratory of Miao Medicine of Guizhou Province, Guiyang 550025, China

**Keywords:** rheumatoid arthritis, Coix Seed Oil, metabolomics, UHPLC-MS, inflammation-associated pathways

## Abstract

Rheumatoid arthritis (RA) is a chronic disease that primarily manifests as symmetrical joint inflammation. Although Coix Seed Oil (CSO) has demonstrated anti-inflammatory effects in RA rat models, its systemic metabolic regulatory mechanisms remain unclear. Therefore, we aimed to investigate whether CSO ameliorates RA by modulating inflammation-associated metabolic pathways. Ultra-High-Performance Liquid Chromatography (UHPLC)-Q Exactive HF-X-MS-based metabolomics was used to profile metabolites in the synovial tissue and serum of complete Freund’s adjuvant (CFA)-induced RA rats. Systematically altered metabolites and their associated pathways were identified using multivariate analysis and pattern recognition. CSO treatment modulated 16 RA-related biomarkers in rat synovial tissues and 12 in the serum, which mainly affected amino acids, arachidonic acids, lipids, sphingolipids, and carnitines. These metabolites were associated with eight perturbed metabolic pathways that were predominantly involved in inflammatory responses. This study demonstrated that CSO has significant anti-RA effects on pharmacodynamic activity and metabolic network regulation. Additionally, inflammation-associated metabolic pathways are closely linked to the therapeutic efficacy of CSO in RA treatment.

## 1. Introduction

Rheumatoid arthritis (RA) is a leading cause of disability worldwide. It is a persistent autoimmune inflammatory condition characterized by synovial overgrowth, ultimately leading to irreversible cartilage and bone degradation [1]. Key mediators of this process include rheumatoid factor (RF), tumor necrosis factor (TNF)-α, interleukin (IL)-1β, and IL-6, which reflect the degree of inflammatory activity and joint damage [2]. RA involves substantial metabolic disturbances, particularly in amino acids, lipids, arachidonic acid, and energy metabolism, which are closely linked to disease progression and inflammatory responses [3,4]. Despite considerable progress in developing target-oriented chemical or biological agents (e.g., nonsteroidal anti-inflammatory drugs, glucocorticoids, IL-1β or TNF-α inhibitors) for treating RA, it remains prominent due to drug side effects, including serious hepatorenal toxicity, cardiovascular disease, vulnerability to severe infection, and overactivation of immune effects during long-term administration [5,6]. Therefore, safer therapeutic agents are required for preventing and managing RA.

Traditional Chinese medicine (TCM) comprises multiple agents that achieve holistic therapeutic outcomes with minimal adverse effects [7]. In China, TCMs have been used to treat refractory diseases, such as RA [8]. The Coix Seed Oil (CSO) is a mixture of oils and lipophilic functional components extracted from *Coix lacryma-jobi* L. var. *ma-yuen* (Roman.) Stapf (commonly known as Coix seed or Yiyiren). The Coix seed (the raw material for CSO) is an important Chinese medicinal herb used to treat RA that is included in the Chinese Pharmacopoeia [9]. The CSO has diverse pharmacological effects, including anticancer, anti-inflammatory, and analgesic properties [10,11]. The CSO exerts multiple effects in RA models, including protection against oxidative damage and pro-inflammatory cytokines [12], suppression of synovial angiogenesis via the HIF-1α/VEGF-A axis [13], inhibition of Th17 cell differentiation [14], and amelioration of collagen-induced arthritis-associated muscle atrophy by rebalancing gut microbiota and the JAK2-STAT3 pathway [15]. Despite its confirmed therapeutic efficacy on RA, the systemic metabolic regulatory mechanisms of the CSO remain unclear.

Assessing the efficacy of TCMs and elucidating their fundamental pharmacological mechanisms are considerably challenging. The therapeutic effects of TCM on RA are due to interactions with a single target, as well as simultaneous interactions with multiple targets [6]. Endogenous small-molecule metabolites reflecting pathological progression or therapeutic intervention have been holistically investigated using metabolomics [16,17], consistent with the whole-system approach of characterizing TCM systemic properties [18]. Metabolomics has been extensively used to evaluate drug toxicity [19], disease diagnosis [20], natural product discovery [21], and the complex mechanisms of TCM [22]. Additionally, it is particularly valuable for identifying disrupted metabolic pathways and evaluating therapeutic interventions [4].

In this study, we aimed to investigate whether the CSO ameliorates RA by modulating inflammation-associated metabolic pathways. We hypothesized that the CSO treatment will restore the levels of key metabolites and reverse the dysregulation of multiple inflammation-linked metabolic pathways.

## 2. Materials and Methods

### 2.1. Materials and Reagents

Complete Freund’s adjuvant (CFA) and celecoxib were purchased from Sigma–Aldrich (St. Louis, MO, USA) and Zoletil 50 was purchased from Virbac Group (Carros, France). A saline solution was obtained from Shandong Hualu Pharmaceutical Co., Ltd. (Liaocheng, China). LC-MS-grade formic acid was purchased from Thermo Fisher Scientific (Waltham, MA, USA) for mass spectrometry. Chromatography-grade acetonitrile and isopropanol were purchased from J.T. Baker (Phillipsburg, NJ, USA). Assay kits for the determination of IL-1β, TNF-α, RF, and IL-6 activities were from Shanghai Chuocai Biotechnology Co., Ltd. (Shanghai, China). The CSO reference extract was purchased from the National Institutes for Food and Drug Control (batch No. 111750-202104). Coix seeds (batch no. 3775929) were obtained from Beijing Tong Ren Tang (Group) Co., Ltd. (Beijing, China) and cultivated in Xingren City, Guizhou Province, China. The CSO was prepared as described previously [10]. The Coix seed powder (600 g) and five times the amount of petroleum ether were placed in a round-bottomed flask. A reflux extraction was conducted for 1 h at 100 °C, and then repeated three times. The solution was filtered, and the filtrates were combined and concentrated under reduced pressure to obtain the CSO.

### 2.2. Analysis of the CSO Using an HPLC Evaporative Light-Scattering Detector (ELSD)

The chemical constituents of CSO were identified using a reference detected with Agilent 1260 Infinity HPLC system (Agilent Technologies, Santa Clara, CA, USA) coupled with an evaporative light scattering detector (ELSD). The CSO (10 mg) was dissolved in 5 mL of mobile phase (acetonitrile to dichloromethane = 65:35, *v*/*v*) and filtered through a 0.22 μm membrane, and the filtrate was used for HPLC analysis. A Zorbax Extend C_18_ column (Agilent Technologies, 4.6 mm × 250 mm, 5 μm) was used for the analysis. The mobile phase was acetonitrile-dichloromethane (65:35, *v*/*v*), flow rate was 1 mL/min, column temperature was 30 °C, and injection volume was 20 μL. For the ELSD, the drift tube temperature was set at 50 °C, nebulizer gas pressure was 50 PSI, and gain was 10.

### 2.3. Experimental Animals

Twenty-four male Sprague–Dawley adult rats weighing 160–180 g were obtained from Changsha Tianqin Biotechnology Co., Ltd. (Changsha, China). All animal studies were approved by the Ethical Review Board of Guizhou University of Chinese Medicine (Guiyang, China, protocol code 20241217001). Rats were housed in cages with free access to water and food. The environment was maintained at 20–25 °C and 40–60% relative humidity, with a 12 h light/dark cycle. The rats underwent a 7-day acclimation period prior to the start of the experiment. Throughout the study, the welfare of all animals was ensured using humane treatment practices, in accordance with the ARRIVE guidelines 2.0.

### 2.4. Animal Handling and Sample Collection

Previous studies investigating three doses of the CSO (low, medium, and high) in collagen-induced arthritis rats demonstrated that a high dose exerted optimal anti-RA efficacy [13]. Therefore, this optimal dose was used in this study. After 1 week of acclimatization, rats were divided into four groups (n = 6 rats/group): Control group (C) rats orally administered 0.3% sodium carboxymethyl cellulose (CMC-Na) at 10 mL/kg; Model group (M) rats orally administered 0.3% CMC-Na at 10 mL/kg; Celecoxib group (XB) rats orally administered celecoxib at 18 mg/kg, and CSO group rats orally administered 8.4 g/kg of CSO. The CFA-induced RA rat model was established as described previously [23]. All groups, except the Control, were administered 0.1 mL intradermal injections of CFA (1 mg/mL) into the right hind paw (subplantar region). Paw thickness measurements in each rat were performed immediately before adjuvant injection to establish a baseline reference. The treatments were administered for 28 days, starting when the first signs of arthritis appeared, such as redness of the paw skin or swelling of the joints. Arthritis symptoms typically appeared 10 days after the CFA injection.

Following the final treatment, the 24 rats were anesthetized with Zoletil 50 (i.p. 40 mg/kg). The right hind paw swelling was measured using Vernier calipers. The degree of paw swelling (mm) was calculated by subtracting paw thickness before inflammation (mm) from that after inflammation (mm) [24]. Blood samples were drawn from the ventral aorta and placed in serum separation tubes. The blood was allowed to clot at 4 °C for 30 min and then centrifuged at 3600× *g* for 10 min at 4 °C to obtain the serum. After blood collection, the rats were cervical dislocated, their skin was removed, and the synovial tissue of the ankle joint was separated for hematoxylin and eosin (H&E) staining and metabolomic examination.

### 2.5. Serum Biochemistry Assays and Histopathology

Serum RF, IL-1β, IL-6, and TNF-α concentrations were measured using a commercially available kit from Shanghai Chuocai Biotechnology Co., Ltd. (Shanghai, China), in accordance with the manufacturer’s protocols.

The synovial tissue was fixed in 4% paraformaldehyde, demineralized, paraffinized, and sectioned into 4 µm thick pieces. Histological changes in the synovial tissue were evaluated using H&E staining. Photomicrographs were obtained after examining the sections under a light microscope (Olympus BX53, Tokyo, Japan). Quantitative analysis of inflammatory cell infiltration was performed using ImageJ software (v1.54, National Institutes of Health, Bethesda, MD, USA). Three randomly selected fields (×100 magnification) were examined in each synovial tissue section. The area of inflammatory cell infiltration was measured as the percentage of positively stained (infiltrated) area relative to the total tissue area. Measurements were conducted independently by two researchers who were blinded to the group allocation and the average values of three fields per section were used for statistical analysis.

### 2.6. Preparation of Synovial and Serum Samples for UHPLC-Q Exactive HF-X-MS Analysis

Metabolites were extracted from processed synovial tissue as previously described [25]. The sample (50 ± 5) mg was placed in a 2 mL centrifuge tube. A hydro-methanolic extraction solvent (500 µL; 80% methanol, *v*/*v*) was added. The sample was subjected to mechanical disruption for 6 min at a temperature of −10 °C using a cryogenic tissue grinder. Ultrasonic-assisted extraction was performed for 30 min under low-temperature conditions (5 °C). After extraction, the samples were chilled at −20 °C for 30 min. After cooling, the sample was centrifuged at 13,000× *g* for 15 min at a 4 °C. After centrifugation, the supernatant was aliquoted into analytical vials using low-volume inserts, prior to chromatographic analysis. The supernatant was then carefully transferred to a vial equipped with an internal cannula for analysis. Aliquots (20 µL) of the supernatant from each sample were combined and used as the quality control (QC).

Serum sample aliquots (100 μL) were placed into 1.5 mL microcentrifuge tubes and 300 μL of a chilled methanol–acetonitrile (1:1) solution was added. After 30 s of vortexing, samples were subjected to ultrasonic extraction (40 kHz, 4 °C, 30 min), 30 min of incubation at −20 °C, and centrifugation (13,000× *g*, 15 min, 4 °C). The resulting supernatant was dried using nitrogen evaporation. The residue was reconstituted with 100 μL of reconstitution solution (acetonitrile to water = 1:1, *v*/*v*), vortexed for 30 s, and subjected to a second round of low-temperature sonication (4 °C, 40 kHz) for 5 min. After 10 min of centrifugation at 13,000× *g* (4 °C), the resulting supernatant was transferred to LC-MS vials with micro-inserts. Equal-volume aliquots (20 μL) from all experimental supernatants were pooled as the quality control and subjected to the same analytical workflow.

### 2.7. UHPLC-Q Exactive HF-X-MS Analysis of the Synovial Tissue and Serum

Chromatographic analysis was conducted using a Thermo Vanquish Horizon UHPLC platform (Waltham, MA, USA, manufactured in 2022) equipped with a Waters HSS T3 analytical column (Waters Corporation, Milford, MA, USA, 100 × 2.1 mm, 1.8 μm). The temperature of the system was maintained at 4 °C for the sample compartment and 40 °C for the column. Chromatographic separation was performed using a binary solvent system, in which mobile phase A comprised aqueous acetonitrile (95:5, *v*/*v*) acidified with 0.1% formic acid and phase B comprised an acetonitrile–isopropanol–water mixture (47.5:47.5:5, *v*/*v*/*v*) with 0.1% formic acid. The following optimized gradient program was used: 0–3 min, 0–20% B; 3–4.5 min, 20–35% B; 4.5–5 min, 35–100% B; 5–6.3 min, 100–100% B; 6.3–6.4 min, 100–0% B; 6.4–8 min, 0–0% B. The injection volume was 3 μL, and the flow rate was 0.4 mL/min.

Mass spectrometry was performed using a Thermo Scientific Q-Exactive HF-X system (Waltham, MA, USA, manufactured in 2022). During UHPLC-MS analysis, electrospray ionization in both positive (ESI^+^) and negative (ESI^−^) modes was carried out. The mass spectrometer was configured to scan masses ranging from *m*/*z* 70 to 1050. The sheath and auxiliary gas flows were maintained at 50 and 13 arbitrary units respectively, using nitrogen as the gas source. The ionization source was maintained at 425 °C (heater) and 325 °C (capillary) to achieve optimal desolvation. The electrospray voltage was optimized to 3.5 kV for ESI^+^ and −3.5 kV for ESI^−^. The S-Lens radiofrequency level of the S-lens was maintained at 50. The collision energy was normalized to three levels: 20%, 40%, and 60%. The resolutions of the full MS and MS^2^ were set to 60,000 and 7500, respectively. Throughout the experiment, lock-mass calibration was performed using official calibration kits from Thermo Fisher Scientific (batch no. 88323).

A QC sample was created to guarantee the stability of the sequence analysis. The UHPLC-MS analytical batch sequence involved random analysis of QC samples. Ten ions were selected to verify the accuracy and repeatability of analytical batch measurements (Table S1). Six duplicate QC samples were used to assess the repeatability of the method. To evaluate injection precision, the QC sample was subjected to six interval analyses. As detailed in Table S1, the relative standard deviations for both the peak area and retention time for all 10 ions remained below 0.5%, indicating the high repeatability and precision of the measurements.

### 2.8. Data Processing

#### 2.8.1. LC-MS Data Preprocessing

The raw data underwent a series of preprocessing steps, including baseline correction, peak detection, area integration, retention time adjustment, and peak alignment, before being imported into the Progenesis QI v3.0 software (Waters Corporation, Milford, MA, USA) for metabolomics data analysis. The final output was a structured dataset containing the chromatographic retention times, precise mass-to-charge ratios, and corresponding peak intensities. A CSV file with the preprocessed data was exported for further multivariate data analysis.

#### 2.8.2. Multivariate Statistical Analysis

Multivariate data modeling was implemented using the commercial SIMCA-P package (v13.0,Umetrics, Umeå, Sweden) following established chemometric protocols. Post-Pareto scaling and unsupervised principal component analysis (PCA) modeling were conducted to elucidate intergroup variations and clustering tendencies within the dataset. To identify group-discriminating metabolites, we constructed an orthogonal partial least squares discriminant analysis (OPLS-DA) model to compare the control and model groups. Discriminatory metabolites were screened using stringent criteria, requiring both variable importance for projection (VIP) > 1 in multivariate modeling and *p*-value < 0.05 in univariate analysis [26]. To account for multiple comparisons, the false discovery rate (FDR) was controlled using the Benjamini–Hochberg method and metabolites with *q* < 0.05 were considered statistically significant. Fold changes were calculated as log_2_FC with 95% confidence intervals. Metabolite annotation was performed using Progenesis QI v3.0, applying a 10 ppm mass tolerance for precursor ions and requiring confirmation using both MS/MS spectral matching and characteristic fragmentation patterns. Compound identification was performed using major metabolomic repositories, including human metabolome database (HMDB), metabolite and tandem MS database (METLIN), and a custom-built database.

#### 2.8.3. Univariate Analysis

The experimental values were represented as mean ± standard deviation (SD). Statistical comparisons were performed using one-way ANOVA followed by Tukey’s post hoc test in IBM SPSS (v20.0, Armonk, NY, USA), with statistical significance defined as *p* < 0.05.

## 3. Results

### 3.1. Chemical Fingerprint Analysis of CSO

**Figure 1:**
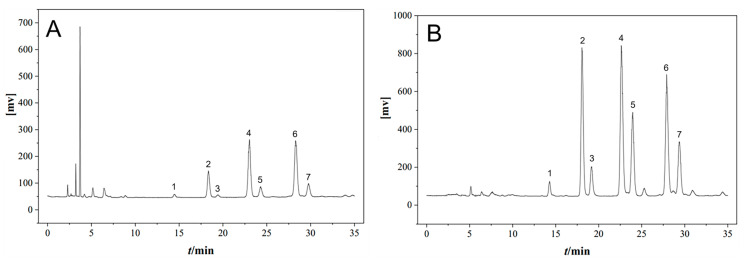
HPLC-ELSD chromatograms of CSO (**A**) and control extract (**B**). Peaks 1, trilinolein; 2, 1,2-linolein-3-linoleic; 3, 1,2-dilinoleoyl-3-palmitoyl-rac-glycerol; 4, 1,2-olein-3-linolein; 5, 1-palmitoyl-2-oleoyl-sn-glycerol; 6, triolein; 7, 1,3-dioleoyl-2-palmitoyl-glycerol.

HPLC-ELSD was used to analyze the CSO, and the typical chromatograms are shown in Figure 1. The chromatograms were confirmed to be consistent with the CSO control extract, the reference characteristic chromatogram specified in the relevant provincial standards, and the elution order reported in the literature [27]. Peaks 1–7 in the chromatogram were identified as: trilinolein (1), 1,2-linolein-3-linoleic (2), 1,2-dilinoleoyl-3-palmitoyl-rac-glycerol (3), 1,2-olein-3-linolein (4), 1-palmitoyl-2-oleoyl-sn-glycerol (5), triolein (6), and 1,3-dioleoyl-2-palmitoyl-glycerol (7).

### 3.2. Paw Swelling and Histopathology of Rats

**Figure 2:**
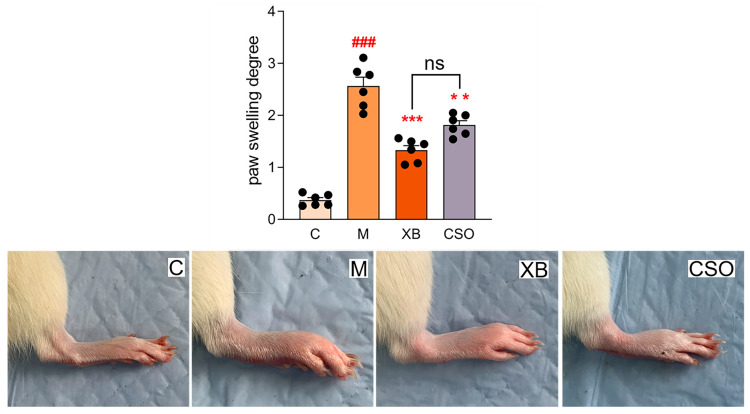
Paw swelling rate of rats. C, control group; M, model group; XB, celecoxib group; CSO, Coix Seed Oil group. mean ± SD (n = 6). ^###^ *p* < 0.001 vs. control; ** *p* < 0.01, *** *p* < 0.001 vs. model group.

A CFA-induced rat model of RA was established to assess the effects of CSO in vivo. Figure 2 illustrates a significantly larger degree of paw swelling (*p* < 0.001) in rats with RA compared with that in controls. After 28 days of treatment with celecoxib or CSO, the degree of paw swelling in rats was significantly reduced compared with that in the model group (*p* < 0.001). Additionally, no significant difference was observed between the CSO and celecoxib groups (*p* = 0.14). These findings suggest that CSO effectively attenuates the progression of arthritis in a rat model of RA.

**Figure 3:**
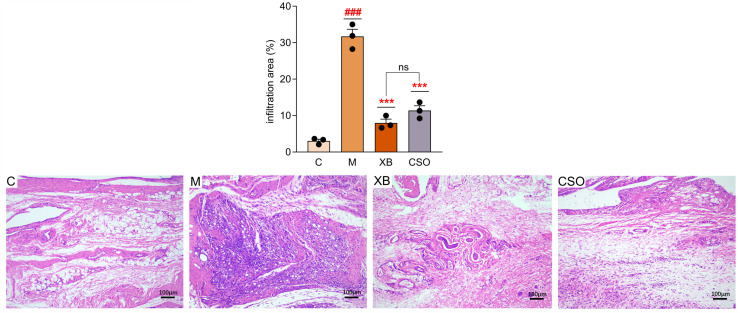
Representative H&E staining images of synovial tissue (×100) and the quantification of inflammatory cell infiltration area (%). C, control group; M, model group; XB, celecoxib group; CSO, Coix Seed Oil group. Data are presented as mean ± SD (n = 3 per group). ^###^ *p* < 0.001 vs. control; *** *p* < 0.001 vs. model group; ns, non-significant.

Histological analysis was used to examine the inflammatory cell infiltration area (%) (Figure 3). The model group had a significantly larger infiltration area than that of the control group (*p* < 0.001). Treatment with CSO and celecoxib significantly reduced the infiltration area compared with that in the model group (both *p* < 0.001), with no statistically significant difference between the CSO and celecoxib groups (*p* = 0.32).

### 3.3. Serum Biochemistry Assays

**Figure 4:**
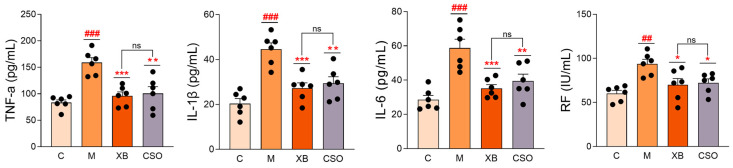
Serum TNF-α, IL-1β, IL-6, and RF levels across experimental groups. C, control group; M, model group; XB, celecoxib group; CSO, Coix Seed Oil group. Data are presented as mean ± SD (n = 6 per group). ^##^ *p* < 0.01, ^###^ *p* < 0.001 vs. control; * *p* < 0.05, ** *p* < 0.01, *** *p* < 0.001 vs. model group; ns, non-significant. Abbreviations: TNF-α, tumor necrosis factor alpha; IL-1β, interleukin 1 beta; IL-6, interleukin 6; RF, rheumatoid factor.

Serum inflammatory marker (TNF-α, IL-1β, IL-6, and RF) levels were significantly higher (*p* < 0.001) in model rats than that in control rats (Figure 4). The 28-day treatment with celecoxib and CSO notably reduced the concentrations of these inflammatory factors compared with those in the model group, with no statistically significant difference between the two treatment groups (*p* > 0.05 for all factors). These results indicate that CSO exerts anti-inflammatory effects comparable to those of celecoxib.

### 3.4. Metabolite Profiles of CFA-Induced RA and CSO-Treated Rats

**Figure 5:**
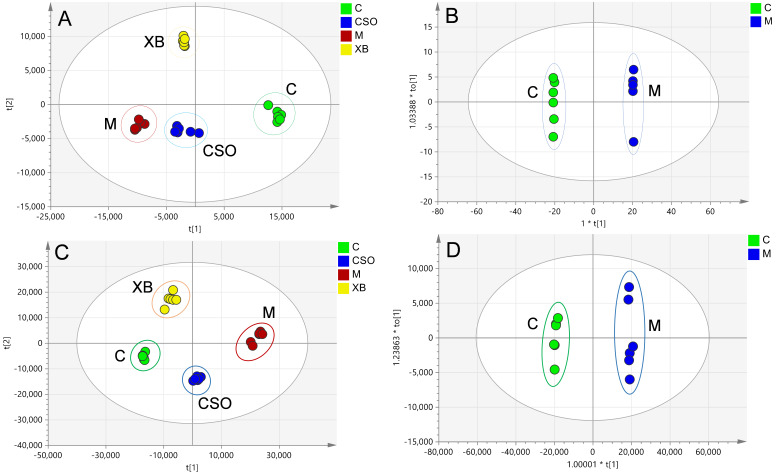
Multivariate statistical evaluation of UHPLC-MS-derived data from synovial tissue (**A**,**B**) and serum (**C**,**D**) samples across different groups. (**A**) PCA scores plot (R^2^X = 0.853, Q^2^ = 0.82); (**B**) OPLS-DA scores plot (R^2^X = 0.883, R^2^Y = 1; Q^2^ = 0.998); (**C**) PCA scores plot (R^2^X = 0.935, Q^2^ = 0.82); (**D**) OPLS-DA scores plot (R^2^X = 0.931, R^2^Y = 0.997; Q^2^ = 0.994). C, control group; M, model group; XB, celecoxib group; CSO, Coix Seed Oil group.

The UHPLC-Q Exactive HF-X platform was used for metabolic characterization of synovial tissues and serum. Figure S1 shows typical chromatographic traces for both sample types. After data preprocessing, the synovial tissue yielded a data matrix of 126 ions in the negative mode and 561 ions in the positive mode, whereas the serum yielded 377 ions in the negative mode and 500 ions in the positive mode. Natural separation of metabolites into several groups was identified using principal component analysis. The metabolites in the control and model groups were distinctly separated (Figure 5A,C), suggesting significant changes in synovial and serum metabolism in rats with RA. The metabolic profiles of rats treated with celecoxib and CSO diverged from those of the model group and were closer to those of the control group. These findings indicated that CSO treatment effectively regulated the metabolic changes induced by RA, consistent with the histological and biochemical analyses.

### 3.5. Identification of RA-Associated Synovial Tissue and Serum Biomarkers

The OPLS-DA (Figure 5B,D), S-plot (Figure S2), and VIP plot (Figure S2) were used to distinguish model and control group metabolites. Sixteen metabolites in the synovial tissue and 12 in serum were identified as RA-associated markers based on VIP > 1 and *p* < 0.05. After FDR correction (*q* < 0.05), all 28 metabolites remained significant. These metabolites, along with their log_2_ FC values and 95% confidence intervals, are presented in Table S2. These metabolites comprised five major classes: amino acids, sphingosines, arachidonic acids, phospholipids, and carnitines (Table 1).

### 3.6. Metabolic Alterations Associated with CFA-Induced RA and the Modulatory Effects of CSO

**Figure 6:**
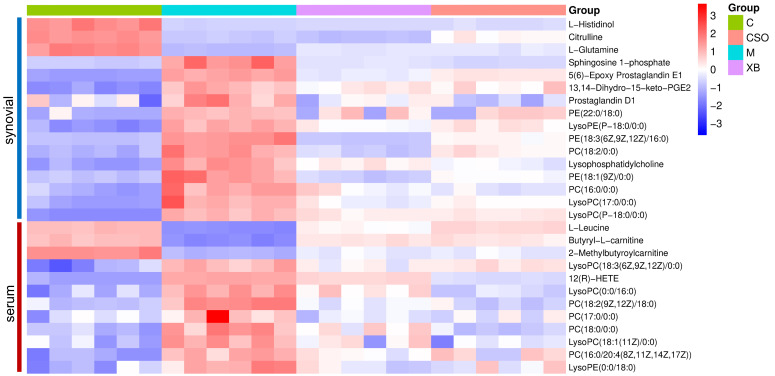
The heat map illustrates the levels of 28 key metabolites in synovial tissue and serum samples. C, control group; M, model group; XB, celecoxib group; CSO, Coix Seed Oil group.

**Figure 7:**
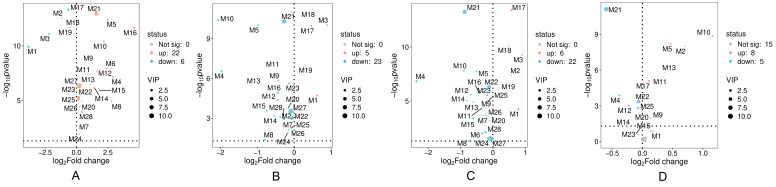
Volcano plot showing the level changes of 28 key metabolites in the synovial tissue and serum of rats in each group. (**A**) C vs. M; (**B**) XB vs. M; (**C**) CSO vs. M; (**D**) XB vs. CSO. C, control group; M, model group; XB, celecoxib group; CSO, Coix Seed Oil group.

To explore the metabolic alterations linked to RA and regulatory effects of CSO, univariate analysis was used to compare the peak areas of the detected metabolites across the experimental groups and perform statistical analyses. Metabolic profiling revealed significantly elevated levels of multiple metabolites in RA rats compared with those in controls, including phospholipids, arachidonic acid derivatives, and sphingosine 1-phosphate (Figure 6 and Figure 7). Conversely, the rats treated with RA exhibited significantly lower levels of amino acids and carnitine derivatives (Figure 7A). Collectively, these findings indicated that CFA-induced RA caused pronounced metabolic disturbances. All detected metabolites exhibited significant normalization of their pathological alterations following celecoxib and CSO administration, compared with those in the model group (Figure 7B,C). Notably, a direct comparison between the two treatment groups (XB vs. CSO, Figure 7D) revealed that 13 of the 28 metabolites showed statistically significant differences (*p* < 0.05), indicating that CSO and celecoxib had distinct regulatory effects on these metabolites.

### 3.7. Dysregulated Metabolic Pathways in RA Rats and CSO Treatments

The Kyoto Encyclopedia of Genes and Genomes database was used to map the metabolic network based on the metabolites associated with the pharmacological modulation and pathological changes identified in our study. In CFA-induced RA rats, significant alterations were observed in eight metabolic pathways: histidine metabolism (P1); arginine biosynthesis (P2); alanine, aspartate, and glutamate metabolism (P3); sphingolipid metabolism (P4); arachidonic acid metabolism (P5); glycerophospholipid metabolism (P6); mTOR signaling (P7); and fatty acid β-oxidation (P8). These pathways showed clear dysfunction in the synovial tissues and serum of RA rats. Importantly, these metabolic impairments were reversed after CSO treatment (Figure 7C).

**Figure 8:**
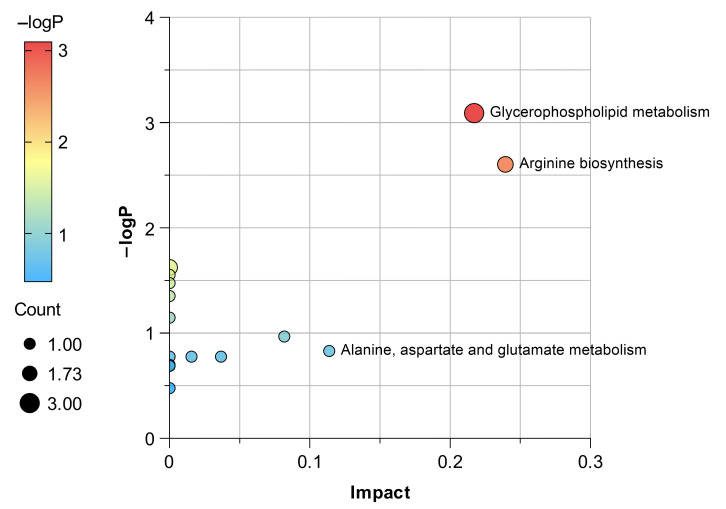
MetPA analysis of the significance of metabolic pathways associated with the 28 key metabolites. A metabolic pathway is represented by each point, and the influence of the metabolic pathway is positively connected with the dot size and color hues.

Furthermore, to evaluate the pathological relevance and therapeutic potential of these metabolic pathways, pathway impact scores were generated using Metabolic Pathway Analysis (MetPA) on MetaboAnalyst 6.0 (Figure 8) [28]. Among the altered pathways, the three pathways with the highest association with pathogenic changes or therapeutic potential (impact > 0.1 [29]) were identified as glycerophospholipid metabolism; alanine, aspartate, and glutamate metabolism; and arginine biosynthesis. These three pathways may be closely linked to the anti-RA effects of CSO in rats with RA.

## 4. Discussion

Both synovial histopathological observation and serum biochemical findings indicated that CSO treatment was associated with the amelioration of RA symptoms. Significant alterations in the metabolic profile occur in patients with RA during its onset and development, primarily involving metabolic disturbances in glucose, lipids, amino acids, arachidonic acid, and carnitine derivatives [3,4]. Therefore, a metabolomics strategy was used to elucidate the therapeutic mechanisms of CSO in RA management. Comprehensive UHPLC-MS-based multivariate analysis revealed that CSO administration effectively reversed metabolic profile disturbances associated with RA progression. This study demonstrated significant metabolic normalization following CSO treatment, indicating its potential to counteract metabolic dysregulation induced by CFA. Metabolic profiling identified 28 disease-associated metabolites that effectively discriminated between RA-induced and healthy rat models. These metabolites were associated with diverse biochemical classes of compounds, including amino acids, sphingosines, arachidonic acids, phospholipids, and carnitines, consistent with previous studies [3,4]. Compared with the model group, the therapeutic administration of CSO exerted significant regulatory effects on these 28 pathological metabolites, which were closely associated with the anti-RA effects of CSO. Integration of pathological and metabolic data revealed that CSO exerts its anti-RA effects through a multi-target mechanism, simultaneously modulating multiple pathological pathways associated with RA progression.

Dysregulated amino acid metabolism significantly affects RA development [30]. In the histidine metabolic pathway, L-histidinol (M1) is a precursor for carnosine synthesis. L-histidinol has many physiological functions, including anti-inflammatory, antioxidative, anti-aging, and mitochondrial damage reduction. It is also involved in apoptosis, autophagy, and immunomodulation [31]. These physiological functions are highly correlated with RA. Citrulline (M2) synergizes with glucosamine and N-acetylglucosamine to exert anti-inflammatory effects in synovial cells. This suggests that combining citrulline with other substances is beneficial for alleviating inflammatory joint diseases [32]. L-glutamine (M3) can be adjunctive agent in the treatment of osteoarthritis by reducing inflammation through suppression of NF-κB and JNK signaling cascades in articular cartilage [33,34]. L-leucine (M17) may exert its anti-inflammatory effects by modulating the mTOR signaling pathway, which could indirectly suppress pro-inflammatory pathways, including NF-κB and MAPK. These pathways serve as central regulators of inflammatory responses, primarily by stimulating pro-inflammatory cytokine production [35]. The reduced levels of L-histidinol, citrulline, L-glutamine, and L-leucine observed in the model group suggest the inhibition of anti-RA physiological functions within the articular cartilage. In this study, CSO treatment effectively reversed the decreased concentrations of these amino acids, indicating that CSO exerted its anti-RA effects by modulating amino acids.

A significant correlation between dysregulated lipid homeostasis and RA pathogenesis was previously established [36]. Clinical investigations have shown that patients with RA frequently present with metabolic lipid abnormalities that actively participate in disease progression and inflammatory responses [37].

Lipids are crucial biological molecules with multiple physiological roles, including energy storage, membrane structure, and cellular signaling [38]. Notably, they function as bioactive mediators of inflammation and immune regulation [39]. Lipid mediators are essential for the pathophysiology of RA. For example, lysophosphatidylcholine modulates immunity, trigger pro-inflammatory mediator synthesis, and amplifies inflammatory responses [40]. In this study, elevated levels of lipids, including phosphatidylethanolamine, lysophosphatidylethanolamine, phosphatidylcholine, and lysophosphatidylcholine, indicated an activated inflammatory response in the joint tissues of CFA-induced RA rats. CSO treatment significantly reduced the lipid levels and alleviated inflammation, highlighting its therapeutic potential for RA-related inflammatory processes.

Sphingolipids are biologically active lipids that control key biological functions such as inflammatory response, apoptosis, proliferation, differentiation, and migration [41]. They are involved in various pathological processes including RA, spondylarthritis, and tumors [42]. Sphingosine 1-phosphate (M4), a synthetic product of sphingosine, is overexpressed in the synovial tissues of patients with RA compared with that in healthy individuals, suggesting its role in the pathophysiology of RA [43]. In our study, CSO administration reversed the elevated sphingosine 1-phosphate levels observed in model rats, suggesting that CSO can improve RA pathophysiology by modulating sphingosine 1-phosphate levels in rats.

The arachidonic acid pathway is complex and yields a variety of metabolites such as prostaglandins and hydroxyeicosatetraenoic acids (HETEs), which contribute to pain and swelling [44]. Metabolomic analysis revealed marked elevations in prostaglandins and HETEs concentrations in both the synovial tissue and serum of the RA model rats. These pro-inflammatory mediators include 5(6)-epoxy prostaglandin E1 (M5), 13,14-dihydro-15-keto-PGE2 (M6), prostaglandin D1 (M7), and 12 (R)-HETE (M21). These metabolites are involved in various inflammatory conditions, notably RA, leading to the gradual deterioration of cartilage and bone [45]. The elevated levels of prostaglandins and HETEs in the model group rats in this study suggested an activated inflammatory response. The CSO treatment significantly lowered prostaglandin levels, thereby alleviating joint pain and swelling.

Carnitine, a naturally occurring compound, acts as an obligatory carrier for long-chain fatty acid import into the mitochondria prior to β-oxidation, thereby providing energy for cellular metabolism [46]. Carnitine exerts potent anti-inflammatory effects by suppressing NF-κB activation [47]. This mechanism leads to significant downregulation of pro-inflammatory cytokines and a reduction in systemic inflammatory markers, such as C-reactive protein, collectively contributing to the amelioration of inflammatory responses [47]. The decreased concentrations of butyryl-L-carnitine (M18) and 2-methylbutyroylcarnitine (M19) in the model group suggest that the inflammatory response was activated in the joint tissues of rats in the model group. Treatment with CSO increased carnitine levels, thereby improving the inflammatory response in joint tissue.

**Figure 9:**
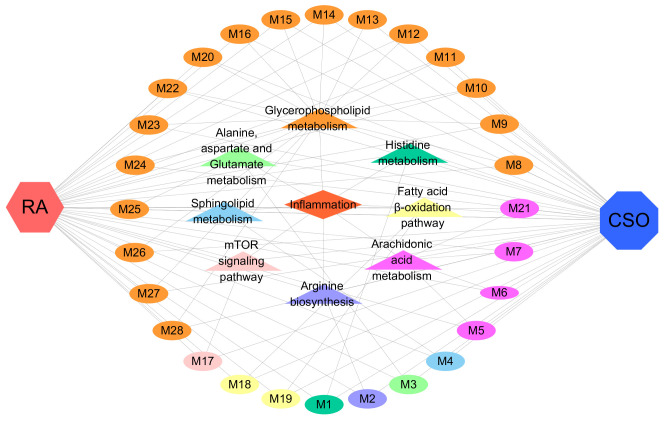
Metabolic network diagram of disordered pathways and metabolites in synovial tissue and serum associated with RA in rats and regulation by CSO. RA, rheumatoid arthritis; CSO, Coix Seed Oil group; M, Metabolite.

Interestingly, disturbances in these pathological markers were predominantly associated with inflammatory responses (Figure 9). CSO treatment significantly ameliorated the dysregulation of these metabolic pathways, suggesting that the suppression of inflammation-associated metabolic pathways may constitute a pivotal mechanism underlying its therapeutic efficacy in RA management. However, the effects of CSO on human RA remain unclear. Future clinical trials and experiments on human cells are needed to verify these findings.

## 5. Conclusions

Through an integrated analysis of serum biochemistry and synovial tissue histopathology, we established the substantial therapeutic efficacy of CSO in alleviating CFA-induced RA in rats. The CSO treatment normalizes the levels of 28 potential biomarkers associated with RA, which modulate perturbations in eight essential metabolic pathways primarily involving amino acid metabolism, lipid metabolism, arachidonic acid metabolism, and fatty acid β-oxidation. These metabolic shifts are predominantly linked to inflammatory responses, suggesting that the suppression of inflammation-associated metabolic pathways may be a key mechanism contributing to the therapeutic efficacy of CSO in RA treatment, consistent with the pharmacodynamic results. CSO and celecoxib differentially regulated 13 of 28 RA-associated metabolites, suggesting pathway-specific differences in their metabolic mechanisms. Consequently, metabolomics combined with pharmacodynamic analysis offers valuable insights into the therapeutic mechanisms of CSO on RA.

## Figures and Tables

**Table 1:** Twenty-eight key differential metabolites identified in rat synovial tissue and serum.

No.	Metabolites	Classification	Pathway
M1	L-Histidinol ^a^	amino acid derivatives	Histidine metabolism (P1)
M2	Citrulline ^a^	amino acid derivatives	Arginine biosynthesis (P2)
M3	L-Glutamine ^a^	amino acid derivatives	Alanine, aspartate and Glutamate metabolism (P3)
M4	Sphingosine 1-phosphate ^a^	sphingosine derivatives	Sphingolipid metabolism (P4)
M5	5 (6)-Epoxy Prostaglandin E1 ^a^	arachidonic acid derivatives	Arachidonic acid metabolism (P5)
M6	13,14-Dihydro-15-keto-PGE2 ^a^	arachidonic acid derivatives	Arachidonic acid metabolism (P5)
M7	Prostaglandin D1 ^a^	arachidonic acid derivatives	Arachidonic acid metabolism (P5)
M8	PE (22:0/18:0) ^a^	phospholipid derivatives	Glycerophospholipid metabolism(P6)
M9	LysoPE (P-18:0/0:0) ^a^	phospholipid derivatives	Glycerophospholipid metabolism (P6)
M10	PE (18:3 (6Z,9Z,12Z)/16:0) ^a^	phospholipid derivatives	Glycerophospholipid metabolism (P6)
M11	PC (18:2/0:0) ^a^	phospholipid derivatives	Glycerophospholipid metabolism (P6)
M12	Lysophosphatidylcholine ^a^	phospholipid derivatives	Glycerophospholipid metabolism (P6)
M13	PE (18:1 (9Z)/0:0) ^a^	phospholipid derivatives	Glycerophospholipid metabolism (P6)
M14	PC (16:0/0:0) ^a^	phospholipid derivatives	Glycerophospholipid metabolism (P6)
M15	LysoPC (17:0/0:0) ^a^	phospholipid derivatives	Glycerophospholipid metabolism (P6)
M16	LysoPC (P-18:0/0:0) ^a^	phospholipid derivatives	Glycerophospholipid metabolism (P6)
M17	L-Leucine ^b^	amino acid derivatives	mTOR signaling pathway (P7)
M18	Butyryl-L-carnitine ^b^	carnitine derivatives	Fatty acid β-oxidation pathway (P8)
M19	2-Methylbutyroylcarnitine ^b^	carnitine derivatives	Fatty acid β-oxidation pathway (P8)
M20	LysoPC (18:3 (6Z,9Z,12Z)/0:0) ^b^	phospholipid derivatives	Glycerophospholipid metabolism (P6)
M21	12 (R)-HETE ^b^	arachidonic acid derivatives	Arachidonic acid metabolism (P5)
M22	LysoPC (0:0/16:0) ^b^	phospholipid derivatives	Glycerophospholipid metabolism (P6)
M23	PC (18:2 (9Z,12Z)/18:0) ^b^	phospholipid derivatives	Glycerophospholipid metabolism (P6)
M24	PC (17:0/0:0) ^b^	phospholipid derivatives	Glycerophospholipid metabolism (P6)
M25	PC (18:0/0:0) ^b^	phospholipid derivatives	Glycerophospholipid metabolism (P6)
M26	LysoPC (18:1 (11Z)/0:0) ^b^	phospholipid derivatives	Glycerophospholipid metabolism (P6)
M27	PC (16:0/20:4 (8Z,11Z,14Z,17Z)) ^b^	phospholipid derivatives	Glycerophospholipid metabolism (P6)
M28	LysoPE (0:0/18:0) ^b^	phospholipid derivatives	Glycerophospholipid metabolism (P6)

^a^ Metabolites were detected in the synovial tissue; ^b^ Metabolites were detected in the serum; M: Metabolite; P: Pathway; Abbreviations: PE, phosphatidylethanolamine; LysoPE, lysophosphatidylethanolamine; PC, phosphatidylcholine; LysoPC, lysophosphatidylcholine; HETE, hydroxyeicosatetraenoic acid; PGE2, prostaglandin E2; mTOR, mammalian target of rapamycin. Other abbreviations are defined in the text or are standard chemical designations.

## Data Availability

All untargeted metabolomic data used in this publication have been deposited to the MetaboLights database with the identifier MTBLS14402 (serum metabolomics) and MTBLS14405 (synovial metabolomics). The complete dataset can be accessed at https://www.ebi.ac.uk/metabolights/MTBLS14402 (accessed on 1 May 2026) and https://www.ebi.ac.uk/metabolights/MTBLS14405 (accessed on 1 May 2026).

## Supplementary Materials

The following supporting information can be downloaded at: https://www.mdpi.com/article/10.3390/cimb48050487/s1.
