# Supplementary material for: Coix Seed Oil Ameliorates Rheumatoid Arthritis by Modulating Inflammation-Associated Metabolic Pathways
Source: Curr Issues Mol Biol. 2026 May 8;48(5):487. doi: 10.3390/cimb48050487 (PMC13206417; doi:10.3390/cimb48050487)
Supplement: Supplementary file 1 [file cimb-48-00487-s001.zip › Supplementary Information.pdf]

Supplementary Information

Table S1. The repeatability and precision of UHPLC-Q Exactive HF-X-MS method validation under the positive ion mode (**A**) and negative ion mode (**B**) using QC sample.

A: positive ion mode

NO.	RT(min)	m/z	metabolite	repeatability		precision	
				RSD(%)$_{RT}$	RSD(%)$_{m/z}$	RSD(%)$_{RT}$	RSD(%)$_{m/z}$
1	0.51	124.0878	L-Histidinol	0.0161	0.0003	0.0220	0.0000
2	2.70	380.4711	Sphingosine 1-phosphate	0.0042	0.0005	0.0160	0.0004
3	6.21	317.2126	13,14-Dihydro-15-keto-PGE2	0.0042	0.0000	0.0510	0.0002
4	6.63	520.3441	PC(18:2/0:0)	0.0074	0.0000	0.0022	0.0001
5	6.85	518.3260	PC(16:0/0:0)	0.0026	0.0000	0.0065	0.0005

B: negative ion mode

NO.	RT(min)	m/z	metabolite	repeatability		precision	
				RSD(%)$_{RT}$	RSD(%)$_{m/z}$	RSD(%)$_{RT}$	RSD(%)$_{m/z}$
1	1.19	181.0392	L-Glutamine	0.0000	0.0003	0.0000	0.0005
2	6.38	838.6118	PE(22:0/18:0)	0.0125	0.0004	0.0000	0.0005
3	6.44	500.2871	LysoPE(P-18:0/0:0)	0.3144	0.0005	0.0000	0.0002
4	6.48	764.5374	PE(P-18:0/18:1(11Z))	0.1067	0.0008	0.3734	0.0005
5	6.92	508.3596	LysoPC(17:0/0:0)	0.0457	0.0013	0.3674	0.0005

The RSD (%)$_{RT}$ and RSD (%)$_{m/z}$ values representing for the RSD of retention time and *m/z* of the selected ions based on UHPLC-Q Exactive HF-X-MS method.

Table S2. Twenty-eight key differential metabolites identified in rat synovial tissue and serum

No.	RT (min)	Adduct ion mass (m/z)	Mass error	Adduct ion	Metabolites	VIP	*p*-value	FDR *q*-value	log_2FC	95% CI
M1	0.51	124.0878	6.79	$M+H-H_2O$	L-Histidinol[a]	2.15	1.18e-10	2.21e-10	-3.82	(-4.14, -3.50)
M2	0.63	176.1039	5.40	M+H	Citrulline[a]	1.17	2.69e-13	9.59e-13	-1.19	(-1.27, -1.11)
M3	1.19	181.0392	4.30	M+Cl	L-Glutamine[a]	1.63	7.86e-12	2.19e-11	-2.20	(-2.36, -2.04)
M4	2.70	380.4711	5.84	M+2Na-H	Sphingosine 1-phosphate[a]	1.86	6.98e-08	9.31e-08	2.86	(2.47, 3.25)
M5	3.08	401.2532	-0.47	$M+CH_3OH+H$	5(6)-Epoxy Prostaglandin E1[a]	1.60	3.81e-13	1.70e-12	2.53	(2.41, 2.65)
M6	6.21	317.2126	4.34	$M+H-2H_2O$	13,14-Dihydro-15-keto-PGE2[a]	1.62	1.14e-08	1.85e-08	2.75	(2.46, 3.04)
M7	6.31	387.2737	-1.07	$M+CH_3OH+H$	Prostaglandin D1[a]	1.10	7.32e-03	7.32e-03	0.18	(0.05, 0.31)
M8	6.38	838.6118	2.50	M+Cl	PE(22:0/18:0)[a]	1.77	1.16e-05	1.62e-05	3.82	(3.41, 4.23)
M9	6.44	500.2871	-8.92	M+Cl	LysoPE(P-18:0/0:0)[a]	1.28	3.65e-09	6.51e-09	1.24	(1.12, 1.36)
M10	6.55	678.4869	1.80	$M+H-2H_2O$	PE(18:3(6Z,9Z,12Z)/16:0)[a]	1.62	3.90e-11	7.80e-11	2.18	(2.05, 2.31)
M11	6.63	520.3441	8.35	M+H	PC(18:2/0:0)[a]	1.06	4.02e-08	5.61e-08	0.91	(0.80, 1.02)
M12	6.70	504.3485	7.13	$M+H-H_2O$	Lysophosphatidylcholine[a]	1.42	1.95e-08	2.99e-08	1.64	(1.50, 1.78)
M13	6.75	480.3120	7.47	M+H	PE(18:1(9Z)/0:0)[a]	1.11	3.35e-07	4.40e-07	1.02	(0.91, 1.13)
M14	6.85	518.3260	8.80	M+Na	PC(16:0/0:0)[a]	1.35	6.86e-07	8.57e-07	1.45	(1.28, 1.62)
M15	6.92	510.3596	8.38	M+H	LysoPC(17:0/0:0)[a]	1.55	1.22e-06	1.46e-06	1.93	(1.79, 2.07)

M16	7.08	508.3802	8.01	M+H	LysoPC(P-18:0/0:0)[a]	2.42	2.23e-12	5.59e-12	4.48	(4.27, 4.69)
M17	0.61	132.1016	-2.15	M+H	L-Leucine[b]	2.21	4.76e-14	3.40e-13	-0.64	(-0.69, -0.59)
M18	1.80	232.1536	-2.84	M+H	Butyryl-L-carnitine[b]	1.13	1.96e-12	5.60e-12	-0.58	(-0.62, -0.54)
M19	2.28	246.1692	-2.89	M+H	2-Methylbutyroylcarnitine[b]	1.10	2.05e-12	6.27e-12	-0.58	(-0.62, -0.54)
M20	5.67	518.3206	-6.85	M+H	LysoPC(18:3(6Z,9Z,12Z)/0:0)[b]	1.14	1.17e-05	1.62e-05	0.40	(0.28, 0.52)
M21	5.88	319.2273	-1.74	M-H	12(R)-HETE[b]	7.60	1.06e-13	5.29e-13	1.63	(1.60, 1.66)
M22	6.03	496.3385	-2.54	M+H	LysoPC(0:0/16:0)[b]	5.38	5.72e-06	8.78e-06	0.12	(0.08, 0.16)
M23	6.13	786.5982	-3.22	M+H	PC(18:2(9Z,12Z)/18:0)[b]	1.51	6.06e-07	7.79e-07	0.20	(0.14, 0.26)
M24	6.20	1019.6998	-3.64	2M+H	PC(17:0/0:0)[b]	1.39	1.19e-02	1.19e-02	0.12	(0.03, 0.21)
M25	6.27	546.3515	-1.56	M+Na	PC(18:0/0:0)[b]	2.16	8.32e-06	1.24e-05	0.12	(0.07, 0.17)
M26	6.45	566.3202	1.70	M+2Na-H	LysoPC(18:1(11Z)/0:0)[b]	1.37	3.89e-05	5.28e-05	0.14	(0.08, 0.20)
M27	6.73	782.5673	-2.75	M+H	PC(16:0/20:4(8Z,11Z,14Z,17Z))[b]	10.49	4.41e-07	5.91e-07	0.15	(0.11, 0.19)
M28	7.49	462.2987	-0.61	M+FA-H	LysoPE(0:0/18:0)[b]	2.03	1.11e-04	1.30e-04	0.43	(0.23, 0.63)

Note: p-values and q-values were calculated based on the raw peak area data using Welch's t-test followed by Benjamini–Hochberg FDR correction. A positive $\log_2FC$ indicates an increase in the model group compared to the control group, while a negative $\log_2FC$ indicates a decrease. [a] Metabolites were detected in the synovial tissue; [b] Metabolites were detected in the serum.

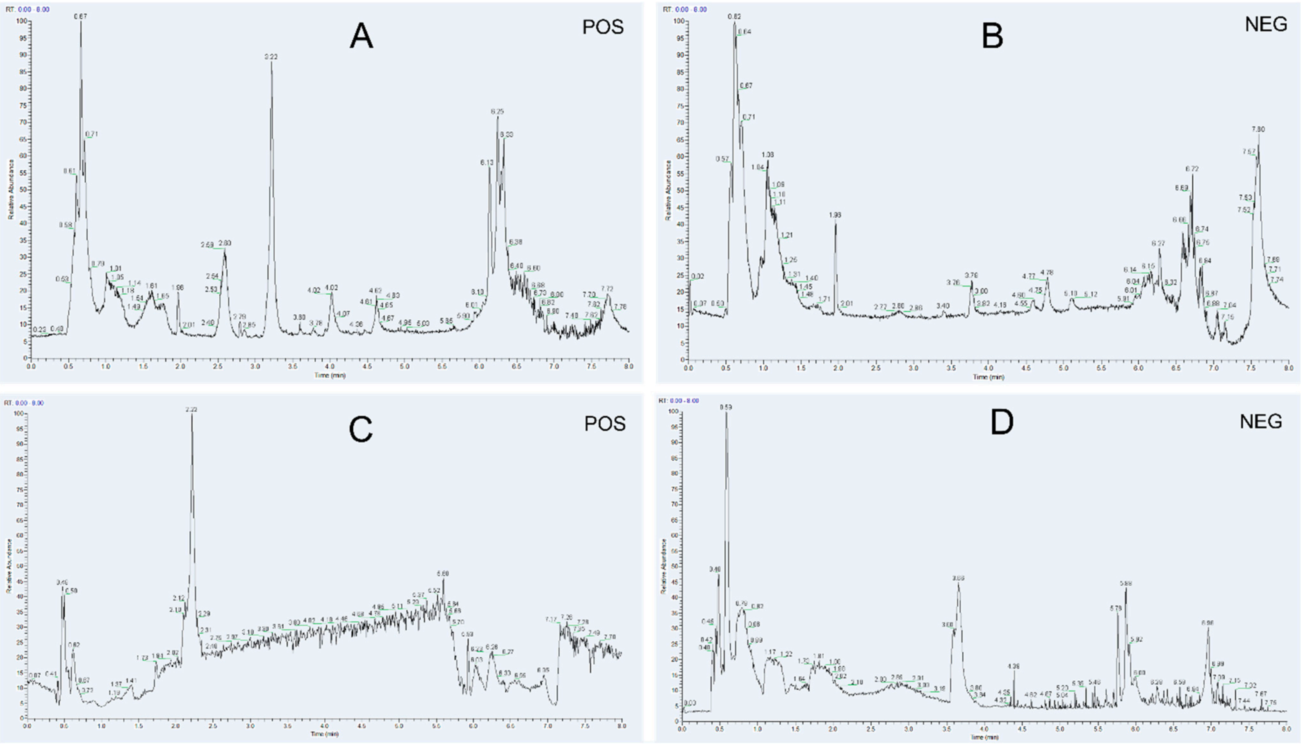

Figure S1. Typical UHPLC-Q Exactive HF-X-MS total ion current chromatograms (TIC) of synovial (A, B) and serum (C, D) samples are shown in positive ion mode (Figure S1A, Figure S1C) and negative ion mode (Figure S1B, Figure S1D).

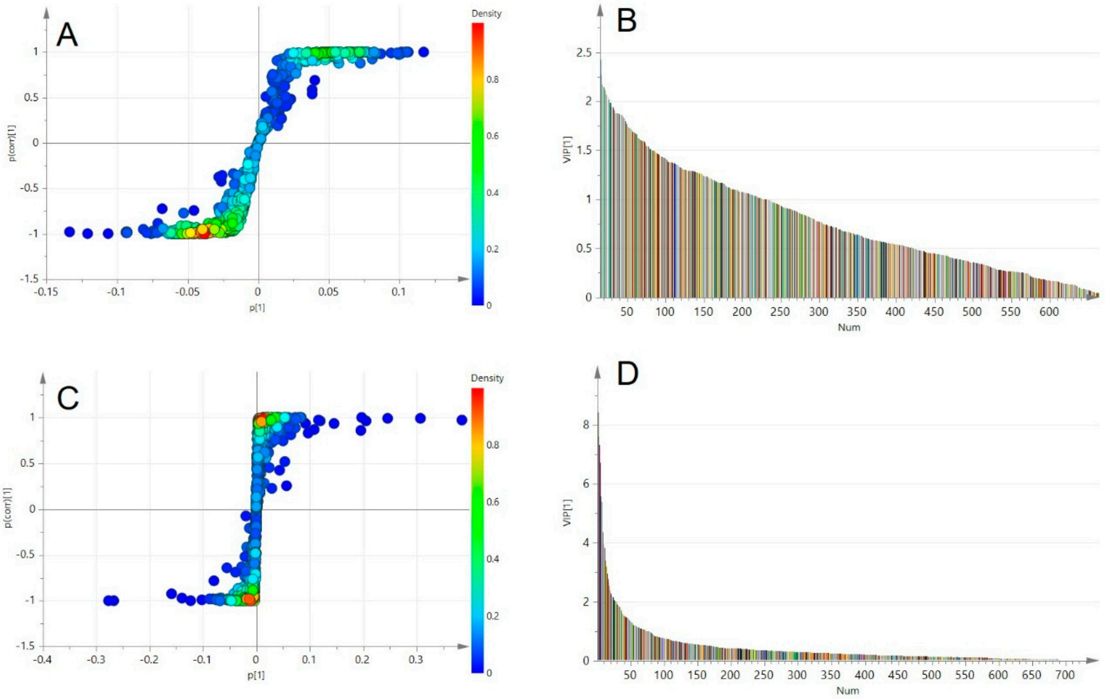

Figure S2. S-plot and VIP-plot of synovial tissue (A and B) and serum (C and D) samples.
